# Deep learning algorithm to evaluate cervical spondylotic myelopathy using lateral cervical spine radiograph

**DOI:** 10.1186/s12883-022-02670-w

**Published:** 2022-04-20

**Authors:** Gun Woo Lee, Hyunkwang Shin, Min Cheol Chang

**Affiliations:** 1 Department of Orthopedic Surgery, Yeungnam University College of Medicine, Yeungnam University, Medical Center, 170 Hyonchung-ro, Namgu, Daegu, 42415 South Korea; 2 Department of Information and Communication Engineering, Yeungnam University, Gyeongsan-si, 38541 Republic of Korea; 3 Department of Rehabilitation Medicine, College of Medicine, Yeungnam University 317-1, Daemyungdong, Namku, Taegu, 705-717 Daegu, Republic of Korea

**Keywords:** Deep learning, Cervical spine, Myelopathy, Radiograph, Artificial intelligence

## Abstract

**Background:**

Deep learning (DL) is an advanced machine learning approach used in different areas such as image analysis, bioinformatics, and natural language processing. A convolutional neural network (CNN) is a representative DL model that is highly advantageous for imaging recognition and classification This study aimed to develop a CNN using lateral cervical spine radiograph to detect cervical spondylotic myelopathy (CSM).

**Methods:**

We retrospectively recruited 207 patients who visited the spine center of a university hospital. Of them, 96 had CSM (CSM patients) while 111 did not have CSM (non-CSM patients). CNN algorithm was used to detect cervical spondylotic myelopathy. Of the included patients, 70% (145 images) were assigned randomly to the training set, while the remaining 30% (62 images) to the test set to measure the model performance.

**Results:**

The accuracy of detecting CSM was 87.1%, and the area under the curve was 0.864 (95% CI, 0.780-0.949).

**Conclusion:**

The CNN model using the lateral cervical spine radiographs of each patient could be helpful in the diagnosis of CSM.

## Background

Cervical spondylotic myelopathy (CSM) is a neurological condition caused by progressive degenerative changes in the intervertebral discs, facets, ligaments, and vertebrae of the cervical spine [1–3]. These degenerative changes directly compress the spinal cord, and CSM is the leading cause of spinal cord injury in adults, accounting for over half of non-traumatic spinal cord injuries [2, 4, 5]. CSM results in motor and sensory deficits, and neuropathic pain, which further cause long-term disability [6]. Early diagnosis of CSM prior to the development of irreversible spinal cord injury is important to achieve a good therapeutic outcome [6].

The diagnosis of CSM is based on cervical spine magnetic resonance imaging (MRI) findings and the corresponding clinical symptoms [7–9]. However, in clinical practice, some patients are reluctant to undergo MRI due to financial concerns. Other than cervical myelopathy, pathologies involving the brain and peripheral nerves can also result in neurological deficits [10]. Therefore, when clinicians have less confidence in the diagnosis of CSM, they may also become hesitant to conduct a cervical MRI. Accordingly, it would be helpful in clinical practice if clinicians can detect CSM on cervical spine radiography. To date, various measurements or findings on cervical radiography have been reported to be correlated with CSM [11–14]. However, the diagnostic accuracy is not sufficiently high.

Machine learning (ML) is a computer algorithm that can automatically learn from data without requiring explicit programming [15]. ML can address the limitations of existing techniques and enable breakthroughs in the field of image analysis. Deep learning (DL) is an advanced ML approach which involves the construction of artificial neural networks with structures and functions similar to those of the human brain using a large number of hidden layers [16]. The DL technique can outperform the traditional ML techniques, and learn unstructured and perceptual image data. A convolutional neural network (CNN) is a representative DL model that is highly advantageous for imaging recognition and classification [17].

In this study, we aimed to develop a CNN model to detect CSM using the lateral cervical spine radiograph of each patient.

## Methods

### Subjects

A total of 207 patients aged ≥20 years who visited the spine center of a university hospital from June 2016 to May 2021 (mean age, 58.6 ± 17.5, M:F = 119:88) were retrospectively recruited for this study. Ninety-six patients (CSM patients) underwent surgery due to symptoms of CSM, and the diagnosis was confirmed through cervical MRI. On the other hand, 111 patients (non-CSM patients) visited the spine center for the management of neck or cervical pain due to herniated cervical disc or cervical stenosis. The absence of CSM was confirmed through cervical MRI. The study protocol was approved by the institutional review board of the university hospital, and was done in accordance with the Declaration of Helsinki. Written informed consent was waived because of the retrospective nature of this study.

### Lateral cervical spine radiographs

One lateral cervical radiograph image of each patient was used as input image data for developing the DL algorithm. The image was taken during the first visit to our spine center. Therefore, the lateral cervical spine radiographs of patients with CSM were obtained prior to cervical spine surgery.

### Deep learning model

**Fig. 1:**
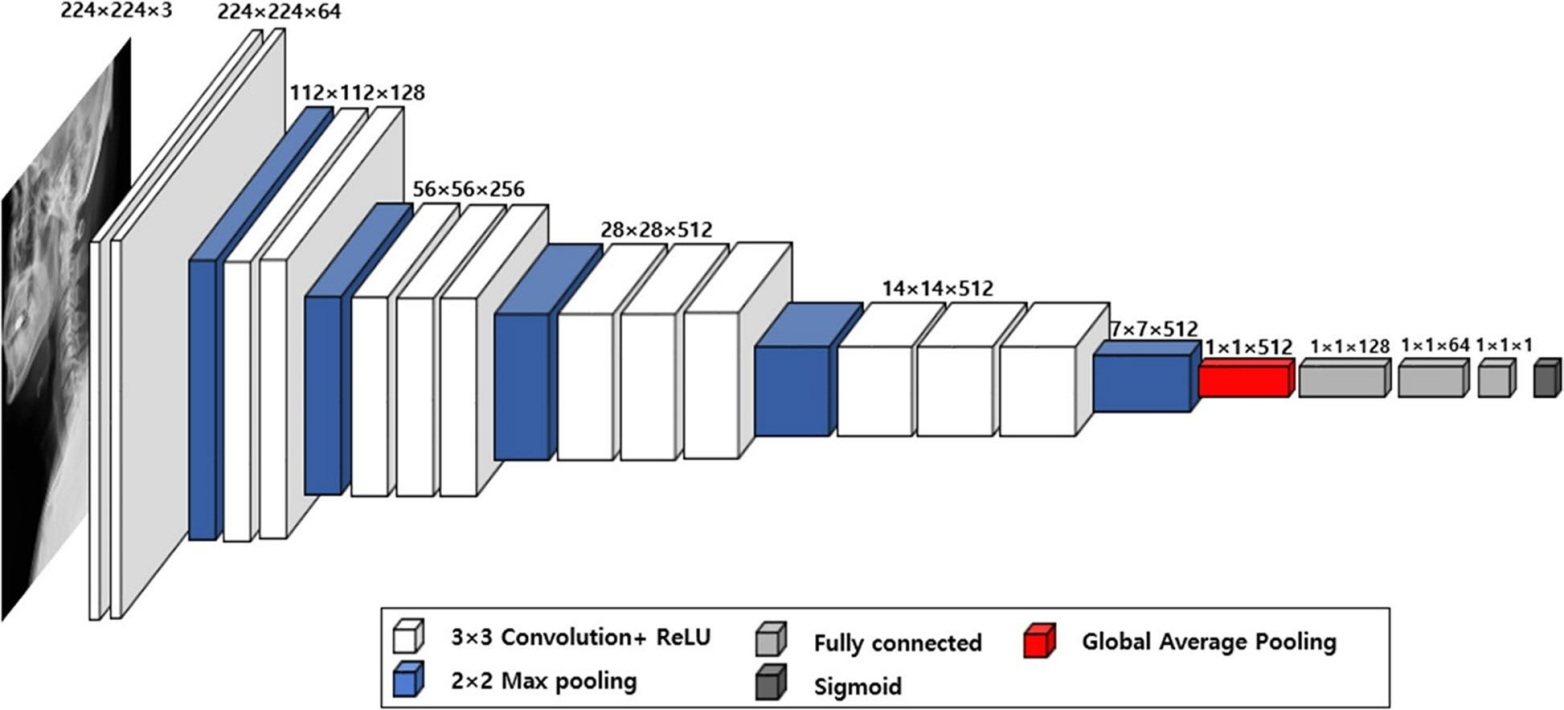
The architecture of convolutional neural network model. ReLU: Rectified Linear Unit

A CNN model was used to predict myelopathy. The model involves 13 convolution layers, a global average pooling layer, and three fully-connected layers. The fully-connected layers (sizes of 128, 64, and 1) were used for classification, and the sigmoid was used for the activation function. The architecture is illustrated in Fig. 1.

The CNN model was implemented in Keras. The Adam optimizer was used with the initial learning rate set to 10^− 5^. The model used pre-trained weights as the initial weights. In addition, data-augmentation methods such as shear, zoom, and width functions were used. The details of the model and its performance are presented in Table 2.

### Experiment

Among the 207 images, 70% (145 images) were randomly selected as training sets, while the remaining 30% (62 images) were assigned to the test set to evaluate the model performance. The details of the dataset configurations are listed in Table 1.

**Table 1:** Dataset configuration

	Train set	Test set
Myelopathy	67	29
Non myelopathy	78	33
Total	145	62

A receiver operating characteristic curve analysis was performed, and the area under the curve (AUC) was calculated using scikit-learn. The 95% confidence interval (CI) for AUC was calculated using the approach used by DeLong et al. [18] The receiver operating characteristic curve analysis and AUC calculation were performed using scikit-learn.

## Results

**Fig. 2:**
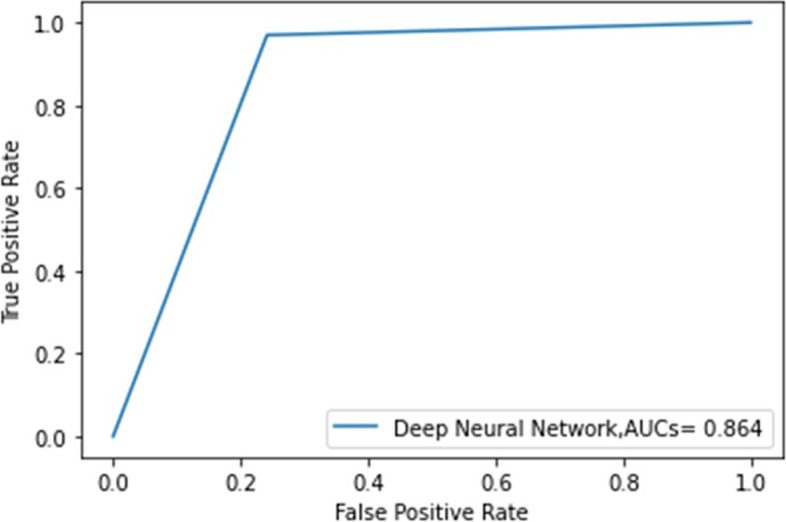
Receiver operating characteristic curve and area under the curve (AUC) for test dataset

The accuracy of classification of CSM and non-CSM with the test dataset using the CNN model was 87.1% (Table 2). Furthermore, the AUC was 0.864 (95% CI, 0.780-0.949) (Fig. 2).

**Table 2:** Performances of the model in diagnosing myelopathy

Model details	Input image size 224 × 224
	Data augmentation (used the zoom, width, and shear function)
	Binary classification with sigmoid activation
	Adam optimizer (the initial learning rate of 10^−5^)
	Batch size 8
Performance	Training accuracy: 92.4%
	Test accuracy: 87.1%
	Test recall: 86.4%
	Test precision: 88.9%
	Test AUC: 0.864 with 95% CI [0.780–0.949]

## Discussion

In this study, a CNN model was developed to determine whether a patient had CSM based on the imaging findings of lateral cervical spine radiograph. The AUC of our model using the test dataset was 0.864. Considering that an AUC of > 0.8 is generally considered excellent, the CNN model that we trained using lateral cervical spine radiograph data can help clinicians diagnose CSM in patients with motor or sensory deficits [19]. We believe that this model may help clinicians in deciding on the necessity of cervical spine MRI.

MRI is the gold standard imaging modality for CSM [20]. However, radiographs are also valuable in the initial approach and presumptive diagnosis of CSM [13, 21]. Some radiographic parameters have been recognized to presume CSM and to determine the necessity of further imaging studies such as MRI. These parameters include cervical canal stenosis with myelopathy based on simple radiographs, including the Torg-Pavlov ratio (TPR), global or segmental sagittal alignment, the presence of abnormal motion (translation or angulation), the detection of ossified lesions such as ossification of the posterior longitudinal ligament (OPLL) and ossification of the yellow ligament (OYL), and others [13, 21–25].

In radiographic images of the cervical spine, the sagittal canal diameter is a critical factor associated with CSM. The narrow spinal canal is linked to canal stenosis and CSM, and is assessed on lateral radiographs using the Torg-Pavlov ratio (TPR). This was measured by the anterior-posterior diameter of the cervical spinal canal divided by the vertebral body width [23, 25]. The TPR method has been widely used for initially presuming CSM or canal stenosis. However, its predictive value varied in previous studies. Moreover, some authors have suggested that TPR may not necessarily correlate with canal stenosis due to its variability and low sensitivity [26]. Cervical alignment is a critical factor in CSM [13, 21, 26]. Cervical myelopathy is highly associated with cervical spondylosis, which contributes to the pathogenesis of cervical myelopathy [13, 21, 26, 27]. Some studies have demonstrated a correlation between the degree of kyphosis of the cervical spine and spinal cord flattening/vascular supply. Sagittal malalignment may be a significant radiographic parameter in predicting CSM on radiographic level. However, only a small number of studies have evaluated the relationship between cervical alignment and myelopathy, and cut-off parameters determining radiographic malalignment have not been elucidated. In addition, previous studies have demonstrated that motion segments adjacent to stiffened, spondylotic segments may exhibit hypermobility and can produce dynamic compression of the spinal cord and myelopathy [21, 28]. However, to date, there is no reported radiographic study on the critical reference of neck motion associated with myelopathy development. The presence of OPLL and OYL increases the likelihood of cervical myelopathy, and cannot provide direct evidence on the presence of CSM. Accordingly, the assessment of the presence of CSM based on radiographs by clinicians’ eyes is not available for use in clinical practice.

Cervical myelopathy is a vexing pathology in terms of disease progression and prognosis. Notably, when it is diagnosed later, the prognosis is generally poor even after surgical treatment. Thus, early presumption and diagnosis are critical in cervical myelopathy. DL is known to address the limitations of the human eye and reveal detailed information that the human eye cannot see. We propose that the DL technique can detect specific evidence of CSM on lateral cervical spine radiographs. A deep neural network (DNN) is characterized by a multi-layer perceptron with multiple hidden layers or a feedforward neural network, which possesses greater ability than the traditional shallow neural network [29]. A CNN is a representative DNN model. It receives input from multiple channels of two-dimensional data and transforms them repeatedly using convolution and pooling operations [17]. These processes allow the extraction of valuable features from the input data. Therefore, CNNs have been used to process image data and recognize image patterns. Our model seemed to recognize the characteristics of lateral cervical spine radiographs of patients with and without CSM, and demonstrated high diagnostic accuracy. Our study is the first to demonstrate the use of DL in detection of CSM on lateral cervical spine radiographs.

## Conclusion

In conclusion, we created a CNN model to diagnose CSM using only one lateral cervical spine radiograph, with an acceptable diagnostic accuracy. However, our study was limited by the small number of subjects with MR images. We recommend further studies with a larger number of subjects to increase the diagnostic accuracy. Future studies should evaluate the accuracy of a CNN model for diagnosing various cervical spinal disorders other than CSM.

## Data Availability

The datasets generated and analyzed during the current study are not publicly available due our privacy policy but are available from the corresponding author on reasonable request.

## Abbreviations

CSM: Cervical spondylotic myelopathy
MRI: Magnetic resonance imaging
ML: Machine learning
CNN: Convolutional neural network
AUC: Area under the curve
OPLL: Posterior longitudinal ligament
OYL: Ossification of the yellow ligament
DNN: Deep neural network
